# Supplementary material for: Reconciling Mining with the Conservation of Cave Biodiversity: A Quantitative Baseline to Help Establish Conservation Priorities
Source: PLoS One. 2016 Dec 20;11(12):e0168348. doi: 10.1371/journal.pone.0168348 (PMC5173368; doi:10.1371/journal.pone.0168348)
Supplement: S1 Dataset — (ZIP) [file pone.0168348.s002.zip › Taxa/Serra Norte/SN_2013/N4E/Lista N4E-29.pdf]

CAVIDADE N4E-0029

Classe	Ordem	Fam/Outros	Gên/Outros	Espécie	S-U	U-S	Zon.
Arachnida	Amblypygi	Phrynidae	*Heterophrynus*	*longicornis*		X	E
Arachnida	Araneae	Araneidae		jovem		X	E
Arachnida	Araneae	Drymusidae		jovem	X		E
Arachnida	Araneae	Pholcidae	*Mesabolivar*	aff. *cambridgei*	X		E
Arachnida	Araneae	Salticidae		sp.2	X		E
Arachnida	Opiliones	Stygnidae	*Protimesius*	*gracilis*	2		E
Arachnida	Pseudoscorpiones	Chernetidae	*Spelaeochernes*	sp.1	X		E
Arachnida	Scorpiones	Buthidae	*Ananteris*	jovem	1		E
Entognatha	Collembola	Entomobryidae		sp.1	X		E
Entognatha	Collembola	Paronellidae		sp.1	X		E
Entognatha	Diplura	Campodeidae		sp.1	X		E
Insecta	Blattodea			jovem	1		E
Insecta	Diptera	Chironomidae		sp.	X		E
Insecta	Diptera	Phoridae	Metopininae	sp.	X		E
Insecta	Diptera	Psychodidae	*Sciopemyia*	*sordellii*	x	X	E
Insecta	Diptera	Tipulidae	Tipulinae	sp.	X		E
Insecta	Heteroptera	Reduviidae	Emesinae	sp.		X	E
Insecta	Heteroptera	Reduviidae	*Zelurus*	sp.	1	X	E
Insecta	Hymenoptera	Diapriidae		sp.1	X		E
Insecta	Hymenoptera	Formicidae	*Pachycondyla*	sp.1	X		E
Insecta	Hymenoptera	Formicidae	*Pheidole*	sp.2	X		E
Insecta	Isoptera			sp.	X		E
Insecta	Lepidoptera	Tineoidea		jovem		X	E
Insecta	Neuroptera	Myrmeleontidae		jovem	X	X	E
Insecta	Orthoptera	Phalangopsidae	*Paraclodes*	jovem	2	X	E
Insecta	Orthoptera	Phalangopsidae	*Phalangopsis*	sp.	2		E
Insecta	Psocoptera	Ptiloneuridae	*Triplocania*	sp.3	X		E
Amphibia	Anura			sp.	2		E
